# A systematic review and meta-analysis of technical aspects and clinical outcomes of botulinum toxin prior to abdominal wall reconstruction

**DOI:** 10.1007/s10029-021-02499-1

**Published:** 2021-09-21

**Authors:** A. S. Timmer, J. J. M. Claessen, J. J. Atema, M. V. H. Rutten, R. Hompes, M. A. Boermeester

**Affiliations:** 1 Department of Surgery, Amsterdam Gastroenterology and Metabolism, Amsterdam Infection and Immunity, Amsterdam University Medical Centers, Location AMC, Suite J1A-228, Meibergdreef 9, 1105AZ Amsterdam, The Netherlands; 2 Department of Anesthesiology, Amsterdam University Medical Centers, Location AMC, Amsterdam, The Netherlands

**Keywords:** Botulinum toxin A, Ventral hernia, Abdominal wall reconstruction

## Abstract

**Purpose:**

To systematically review technical aspects and treatment regimens of botulinum toxin A (BTA) injections in the lateral abdominal wall musculature. We also investigated the effect of BTA on abdominal muscle- and hernia dimensions, and clinical outcome.

**Methods:**

PubMed, EMBASE, CENTRAL, and CINAHL were searched for studies that investigate the injection of BTA in the lateral abdominal wall muscles. Study characteristics, BTA treatment regimens, surgical procedures, and clinical outcomes are presented descriptively. The effect of BTA on muscle- and hernia dimensions is analyzed using random-effects meta-analyses, and exclusively for studies that investigate ventral incisional hernia patients.

**Results:**

We identified 23 studies, comprising 995 patients. Generally, either 500 units of Dysport^®^ or 200–300 units of Botox^®^ are injected at 3–5 locations bilaterally in all three muscles of the lateral abdominal wall, about 4 weeks prior to surgery. No major procedural complications are reported. Meta-analyses show that BTA provides significant elongation of the lateral abdominal wall of 3.2 cm per side (95% CI 2.0–4.3, *I*^2^ = 0%, *p* < 0.001); 6.3 cm total elongation, and a significant but heterogeneous decrease in transverse hernia width (95% CI 0.2–6.8, *I*^2^ = 94%, *p* = 0.04). Furthermore, meta-analysis shows that BTA pretreatment in ventral hernia patients significantly increases the fascial closure rate [RR 1.08 (95% CI 1.02–1.16, *I*^2^ = 0%, *p* = 0.02)].

**Conclusion:**

The injection technique and treatment regimens of botulinum toxin A as well as patient selection require standardization. Bilateral pretreatment in hernia patients significantly elongates the lateral abdominal wall muscles, making fascial closure during surgical hernia repair more likely.

**Study registration:**

A review protocol for this meta-analysis was registered at PROSPERO (CRD42020198246).

**Supplementary Information:**

The online version contains supplementary material available at 10.1007/s10029-021-02499-1.

## Introduction

Ventral incisional hernia is a common complication after laparotomy. It is estimated that in the United States alone, more than 500,000 hernia repairs are performed annually [1, 2]. Incisional hernia repair is therewith one of the most frequently performed surgical procedures. The aim during incisional hernia repair is to achieve tension-free, mesh reinforced midline fascial closure. Mesh reinforcement offers direct support to the reconstruction and reduces the lateral force (sheer stress) on the repair. In patients with a large hernia width, chronically retracted lateral abdominal wall muscles, and irreducible visceral content in the hernia, midline closure can be difficult or even impossible. A bridged hernia repair however should be avoided whenever possible, as this is associated with unacceptable high hernia recurrence rates [3, 4]. Surgical component separation techniques (CST), frequently performed during abdominal wall reconstruction (AWR), increase abdominal wall pliability and facilitate fascial medialization. Component separation techniques are associated with an increased risk of surgical site morbidity, such as infection, wound dehiscence, and seroma formation, which vary between the different techniques [4, 5].

Botulinum toxin A (BTA) is a neurotoxin derived from the bacteria clostridium botulinum. When injected into muscle tissue, it causes a temporary flaccid muscle paralysis [6]. Its paralyzing effect peaks around 2–4 weeks after injection and decreases in the months after. The preoperative chemical paralysis of the lateral abdominal wall using BTA—first described in 2009—has the same goal as surgical CST, namely increasing pliability and reducing the lateral force in an effort to facilitate midline closure [7]. This technique is far less invasive and has an effect on all the muscle layers that are injected, instead of only the muscle layer that is transected from the others. Since then, several studies have described the use of BTA in different clinical scenarios regarding AWR. It is proposed that preoperative chemical paralysis of the lateral abdominal wall muscles—with BTA—increases abdominal wall compliance and therewith facilitates fascial medialization, and possibly even precludes the need to perform CST [8–10]. Despite initial positive reports, the use of BTA for this new indication is currently off-label and consensus on the indications, technical strategies, and outcome measurements are lacking. The main objective of this study was to present an overview of the indications, technical aspects, and treatment regimens of BTA injections regardless of the clinical scenario. Specifically for patients with a ventral incisional hernia, we investigated how BTA affects abdominal muscle- and hernia dimensions, and present clinical outcomes.

## Methods

### Registration

This systematic review and meta-analyses are conducted following the Preferred Reporting Items for Systematic Reviews and Meta-Analyses (PRISMA) statement [11]. A concise description of the study protocol was registered to the International Prospective Register of Systematic Reviews (PROSPERO registration number CRD42020198246).

### Study eligibility

Studies that describe the injection of BTA into the lateral abdominal wall muscles in ≥ 5 patients were eligible for inclusion. Eligibility was regardless of clinical scenario. Studies reporting in any other than European language, non-human studies, and conference abstracts were excluded.

For the second objective, we selected only those studies that describe the intramuscular injection of BTA in patients with a ventral incisional hernia and report computed tomography (CT) measures obtained both before- and after BTA treatment. Studies that describe patients with an abdominal wall defect other than a ventral hernia were analyzed only for other endpoints (BTA techniques and regimens). Studies that use concomitant progressive pneumoperitoneum (PPP) besides BTA were analyzed separately.

### Information source

The PubMed (Medline), EMBASE (Ovid), Cochrane Central Register of Controlled Trials (CENTRAL), and CINAHL (EBSCO) databases were searched without date or language restrictions. The search was finalized on March 23, 2021. The search strategy includes the free text and index terms: abdominal hernia, ventral hernia, midline hernia, incisional hernia, botox, and botulinum toxin (see Supplementary Information 1, which demonstrates the full PubMed search strategy).

### Study selection

Two reviewers (AST and JJMC) independently considered the studies—that were identified by the search strategy—for eligibility. If title and abstract showed potential eligibility or eligibility was in doubt, the full text was assessed for final decision. If studies showed ineligibility during full-text assessment, the reasons were documented. Groups that published more than one eligible study were contacted to ensure that only the most recent study describing the same (subgroup of) patients was included. Discrepancies were resolved through discussion.

### Data collection

Data were collected in duplicate by two reviewers (AST and JJMC) using a predefined data extraction form. Only aggregate data from the original manuscripts were collected. After independent data collection, data were compared, checked for, and discrepancies were resolved through discussion.

### Data items

Study characteristics: year of publication, study design, sample size, and participant specification.

BTA characteristics: indication, type of BTA, dosage, injected volume, number of injections, injected muscles, radiological guidance, assessment of intramuscular distribution, direct complications, use of concomitant PPP, and timing prior to surgery. CT measures: transverse hernia width, lateral abdominal wall muscle length (measured along the inner surface from the quadratus lumborum to the rectus abdominis) and thickness (measured from the inner side of the transversus abdominis to the outer side of the external oblique), intra-abdominal width, intra-abdominal volume, hernia volume, and loss of domain. Surgical outcomes: type of surgical repair, CST, use of mesh, fascial closure rate, postoperative pain medication, follow-up, and hernia recurrence.

### Quality assessment

The risk of bias in the RCT was assessed using the revised Cochrane risk of bias tool (RoB 2) [12]. The body of evidence was assessed using the Newcastle–Ottawa Quality Assessment Form [13].

### Data analysis

Study- and patient specifications, BTA characteristics, and surgical data are presented descriptively. Numerical data are presented as mean with standard deviation (SD) or median with interquartile range (IQR), depending on normality. Normality was checked by plotting a frequency distribution. Categorical data are presented as percentage. Abdominal muscle- and hernia dimensions obtained from before and after treatment are pooled and expressed in mean difference (MD). If studies report the average change in muscle length for both sides rather than the change per side, this average number was used for both sides in the summary estimates. Data from studies that performed additional PPP are reported separately. Considering heterogeneity in patient characteristics, surgical techniques, and different follow-up times, pooling of clinical outcomes was inappropriate. Pooled effect estimates with corresponding 95% confidence intervals (95% CI) were calculated using a random-effects model (inverse variance), with *p* < 0.05 considered statistically significant. Statistical analysis was performed using Review Manager (RevMan Version 5.3. Copenhagen: The Nordic Cochrane Centre, The Cochrane Collaboration, 2014).

## Results

### Study selection

**Fig. 1:**
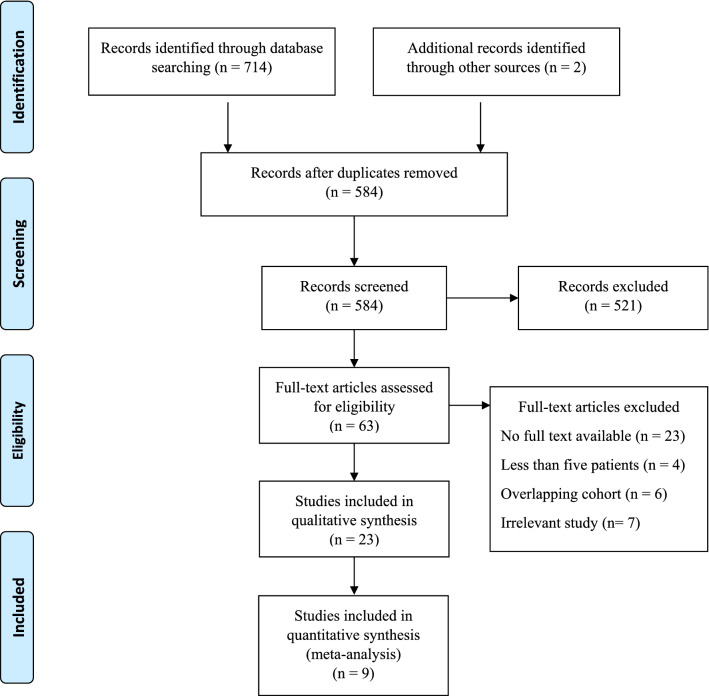
PRISMA systematic review flow diagram

The primary search yielded 714 results. After removal of duplicates, titles and abstracts of 584 studies were screened. Following full-text assessment of 63 studies, 23 studies were included (Fig. 1).

### Study- and patient characteristics

Study- and patient characteristics are presented in Table 1. We identified one RCT [14], two prospective comparative studies [15, 16], three retrospective comparative studies [17–19], six prospective cohort studies [7, 20–24], and eleven retrospective cohort studies [25–35], all published between 2009 and 2021. Eighteen studies describe the injection of BTA to optimize patients prior to ventral hernia repair and one study [19] performs intraoperative BTA injections during open hernia repair. Two studies [14, 32] describe the use of BTA in patients with an open abdomen following damage-control laparotomy (DCL), one study [24] describes patients with giant inguinoscrotal hernia, and one study [34] describes a combination of ventral, flank and inguinal hernia. Combined, the 23 studies include 995 patients of which 664 have been treated with BTA. The median defect width from 15 studies [7, 16–23, 25–30] that exclusively describe patients with a midline ventral hernia is 13.9 cm (IQR 11.4–14.7). Seven [15, 18, 24–26, 30, 31] of 20 studies that inject BTA prior to a hernia repair report a specific indication for the use of BTA. These indications vary between expected difficulty obtaining midline closure to a specific hernia width with or without loss of domain.

**Table 1:** Study- and patient characteristics

References	Patients treated with BTA (in study)	Study design	Patient specification	Mean age (year)	Midline defect (%)	Mean defect width (cm)	Specific indication for BTA
1a: studies investigating BTA specifically for ventral incisional hernias							
Blaha [19]	19 (41)	Comparative cohort (r)	Symptomatic VIH	54.3	n.r.	8.5	n.r.
Bueno-Lledó [25]	100 (100)	Cohort (r)	VIH with LOD	59.4	82%	16.1	VIH ≥ 12 cm and LOD < 20%
Bueno-Lledó [15]	40 (80)	Comparative cohort (p)	VIH between 11 and 17 cm	54.5	100%	14.9	VIH 12–18 cm and LOD < 20%
Catalan-Garza [26]	36 (36)	Cohort (r)	Complex VIH ≥ 10 cm or LOD > 20%	60.9	n.r.	13.9	VIH ≥ 10 cm and LOD > 20%
Chan [27]	7 (12)	Cohort (r)	Complex VIH	72.0	58%	n.r.	n.r.
Cháves-Tostado [28]	14 (14)	Cohort (r)	VIH > 20 cm	58.0	62%	14.6	n.r.
Deerenberg [18]	75 (220)	Comparative cohort (r)	Massive VIH	61	n.r.	14.1	Clinical assessment (a)
Elstner [20]	32 (32)	Cohort (p)	Complex VIH	58.0	72%	12.3	n.r.
Elstner [16]	46 (46)	Comparative cohort (p)	Complex VIH	63.0	n.r.	11.4	n.r.
Farooque [21]	8 (8)	Cohort (p)	Complex VIH ≥ 6 cm or LOD ≥ 15%	62.0	88%	11.0	n.r.
Hernández López [29]	36 (36)	Cohort (r)	VIH 10–15 cm	52.0	n.r.	n.r.	n.r.
Ibarra-Hurtado [7]	12 (12)	Cohort (p)	Abdominal hernia after open abdomen treatment	34.3	100%	13.9	n.r.
Ibarra-Hurtado [22]	17 (17)	Cohort (p)	Abdominal hernia after open abdomen treatment	34.8	100%	14.7	n.r.
Kohler [33]	34 (34)	Cohort (r)	VIH	63.0	85%	12.2	n.r.
Nielsen [30]	37 (37)	Cohort (r)	Large VIH	59.5	92%	12.1	Clinical suspicion of LOD or (b)
Palmisano [23]	38 (38)	Cohort (p)	VIH 10–15 cm	55.3	n.r.	11.2	n.r.
Tashkandi [35]	13 (41)	Cohort (r)	VIH with LOD	58.0	n.r.	16.0	n.r.
Yurtkap [31]	20 (23)	Cohort (r)	Abdominal hernia > 12 cm	65.0	n.r.	n.r.	VIH 14–22 cm without LOD
Zendejas [17]	22 (88)	Comparative cohort (r)	VIH	61.8	n.r.	n.r.	n.r.
1b: studies investigating BTA for indications other than ventral incisional hernia							
Canario [34]	8 (8)	Cohort (r)	Ventral, flank or inguinal hernia	50.1	n.r.	n.r.	n.r.
Tang [24]	8 (8)	Cohort (p)	Giant inguinoscrotal hernia	65.0	n.a	5.4	LOD > 20%
Zielinski [32]	18 (18)	Cohort (r)	Open abdomen following DCL	66.0	100%	n.r.	n.r.
Zielinski [14]	24 (46)	RCT	Open abdomen following DCL	60.0	100%	n.r.	n.r.

*(r)* retrospective, *(p)* prospective, *RCT* randomized-controlled trial, *VIH* ventral incisional hernia, *LOD* loss of domain, *DCL* damage-control laparotomy

(a) Clinical suspicion that fascial closure would be unlikely, even with CST

(b) Expected difficulty obtaining midline closure intraoperatively

### BTA characteristics

Botulinum injection techniques and treatment regimens are presented in Table 2. Sixteen studies [14, 16–21, 24, 26–32, 34] use onabotulinumtoxin A (Botox^®^, Allergan, Dublin, Ireland) with a dose varying between 100 and 300 units. Five studies [7, 15, 22, 25, 35] use 500 units of abobotulinumtoxin A (Dysport^®^, IPSEN, Boulogne-Billancourt, France), one study [23] uses 100 units of incobotulinumtoxinA (Xeomin^®^ Merz Pharmaceuticals GmbH, Frankfurt, Germany), and one study uses 400 units of BTA but does not specify the brand [33]. The median volume in which BTA is diluted for injection is 150 mL (IQR 52.5–150). Thirteen studies [14, 16–18, 20, 21, 23, 24, 26, 31, 32, 34, 35] perform three injections bilaterally, located on the anterior axillary line, between the costal border and iliac spine. Four studies [7, 15, 22, 28] perform five injections bilaterally, two on the anterior axillary line and three over the external oblique muscle. One study [30] uses three-to-five injection bilaterally. Fifteen studies [14, 15, 17, 18, 20, 21, 24–26, 30–35] inject all three muscles (external oblique, internal oblique and transversus abdominis), whereas two studies [22, 27] spare the transversus abdominis from injection. One study [16] specifically investigates the standard three-layer against the selective two-layer muscle injection. In the elective setting, BTA is generally injected about 4 weeks preoperatively (range 0–6 weeks). In patients with an open abdomen, BTA is injected within 2 days following the DCL. Injections are predominantly performed under ultrasonographic (US) guidance. Two studies [15, 25] use additional electromyographic guidance (EMG), two studies [7, 28] exclusively use EMG, one study [18] describes guidance with low dose CT, and one study [19] injects BTA intraoperative under direct visualization. None of the studies report procedural visualization of intramuscular spread. Sixteen studies [14–17, 20–22, 24–27, 29–32, 34] report to have observed no direct major complications following the injections. Four studies [16, 20, 21, 30] do however describe that patients commonly report pain immediately after the injection, a feeling of abdominal distension or bloating, weaker cough or sneeze, and back pain. Not one of these side effects required intervention. Additional PPP is performed in five studies [24, 25, 31, 33, 35].

**Table 2:** BTA injection techniques and treatment regimens

References	Type BTA, units (IE)	Volume injected (mL)	Injections (per side)	Muscles injected	Timing prior to surgery	Radiological guidance	Additional PPP
2a: studies investigating BTA specifically for ventral incisional hernias							
Blaha [19]	Botox^®^, 200	30	n.r.	EO, IO, TA, RA	Intraoperative	None (direct visualization)	No
Bueno-Lledó [25]	Dysport^®^, 500	50	n.r.	EO, IO, TA	38 days	US + EMG	Yes (100%)
Bueno-Lledó [15]	Dysport^®^, 500	50	5	EO, IO, TA	34 days	US + EMG	No
Catalan-Garza [26]	Botox^®^, 300	150	3	EO, IO, TA	6 weeks	US	No
Chan [27]	Botox^®^, 200	n.r.	n.r.	EO, IO	30 days	US	No
Cháves-Tostado [28]	Botox^®^, 100	n.r.	5	n.r.	40 days	EMG	No
Deerenberg [18]	Botox^®^, 200 or 300	100 or 150	3	EO, IO, TA	4 weeks	US or CT	No
Elstner [20]	Botox^®^, 300^a^	150	3	EO, IO, TA	1–4 weeks	US	No
Elstner [16]	Botox^®^, 200	100	3	EO, IO or EO, IO, TA	2–4 weeks	US	No
Farooque [21]	Botox^®^, 300	150	3	EO, IO, TA	2 weeks	US	No
Hernández López [29]	Botox^®^, n.r.	n.r.	n.r.	n.r.	4 weeks	n.r.	No
Ibarra-Hurtado [7]	Dysport^®^, 500	n.r.	5	n.r.	4 weeks	EMG	No
Ibarra-Hurtado [22]	Dysport^®^, 500	5	5	EO, IO	4 weeks	US	No
Kohler [33]	Not specified, 400	n.r.	n.r.	EO, IO, TA	26 days	US	Yes (6%)
Nielsen [30]	Botox^®^, 300	60 or 150	3 or 5	EO, IO, TA	32 days	US	No
Palmisano [23]	Botox^®^, 200 or Xeomin^®^, 100	n.r.	n.r.	n.r.	4 weeks	US	No
Tashkandi [35]	Dysport^®^, 500	180	3	EO, IO, TA	3–6 weeks	US	Yes (100%)
Yurtkap [31]	Botox^®^, 300	150	3	EO, IO, TA	45 days	US	Yes (74%)
Zendejas [17]	Botox^®^, 300	150	3	EO, IO, TA	Range 0–19^b^	US	No
2b: studies investigating BTA for indications other than ventral incisional hernia							
Canario [34]	Botox^®^, 300	150	3	EO, IO, TA	34 days	US	No
Tang [24]	Botox^®^, 100	50	3	EO, IO, TA	n.r.	US	Yes (100%)
Zielinski [32]	Botox^®^, 300	150	3	EO, IO, TA	< 1 day after DCL^c^	US	No
Zielinski [14]	Botox^®^, 150	300	3	EO, IO, TA	1.8 days after DCL	US	No

*Dysport*^®^ abobotulinumtoxinA, *Botox*^®^ OnabotulinumtoxinA, *Xeomin*^*®*^ IncobotulinumtoxinA, *EO* external oblique muscle, *IO* internal oblique muscle, *TA* transversus abdominis muscle, *RA* rectus abdominis, *DCL* damage-control laparotomy, *US* ultrasound, *EMG* electromyography, *PPP* progressive pneumoperitoneum, *n.r.* not reported

^a^Most patients received a total dose of 300 Botox^®^ or equivalent dose of Dysport^®^

^b^Due to logistical and patient clinical issues, nine patients (41%) had it a median of 6 days (range 1–19 days) prior to surgery, and 13 (59%) had it performed on the same day as surgery

^c^Nine patients (50%) underwent BTA injections within 24 h of their open abdomen procedure. Timing in the other nine patients is not mentioned

### Quality assessment

The body of evidence was rated good in three studies [15, 17, 18], fair in one study [14], and poor in nineteen studies [7, 16, 19–35], ranging from three to seven stars using the Newcastle–Ottawa ranking scale (see Supplementary Information 2, which demonstrates the full body of evidence assessment). Most studies lack a control group for comparison. A follow-up time shorter than 1 year was deemed inadequate in studies that describe patients with ventral hernia. Additional risk of bias assessment of the RCT showed ‘some risk of bias’ in the selection of reported results, and low risk of bias in all other domains.

### Effect of BTA on muscle- and hernia dimensions

#### Length of the lateral abdominal wall

**Fig. 2:**
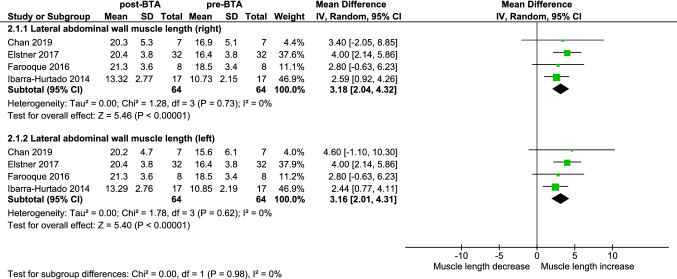
Forest plot of the difference in length of the lateral abdominal wall muscles in centimeters before and after BTA (without PPP). *2.1.1* right-ride muscles, *2.1.2* left-side muscles

Meta-analysis of four studies [20–22, 27] shows that BTA significantly increases the lateral abdominal wall muscle length by 3.2 cm on each side (95% CI 2.0–4.3, *p* < 0.001, *I*^2^ = 0%); 6.3 cm total elongation (Fig. 2).

Elstner et al. [16] included 46 patients of which 23 underwent standard three-layer injection. The other 23 patients underwent selective injection into the external oblique and internal oblique muscle only, thus sparing the transversus abdominis muscle. Baseline demographics including mean hernia width did not differ between both groups. Repeated CT imaging 2–4 weeks after injection shows a significant increase in length of the lateral abdominal wall of 3.8 cm per side, however, comparable among the standard three-layer and the selective two-layer groups (*p* = 0.365).

#### Thickness of the lateral abdominal wall

Ibarra-Hurtado et al. [22] investigate the effect of BTA on the thickness of the three lateral abdominal wall muscles together, and find a significant decrease in combined muscle thickness of 1.0 cm on both sides (*n* = 17, *p* < 0.001). Of note, muscles thickness is measured in the horizontal plane and not perpendicular to the direction of the muscle fibers.

#### Transverse hernia width

**Fig. 3:**
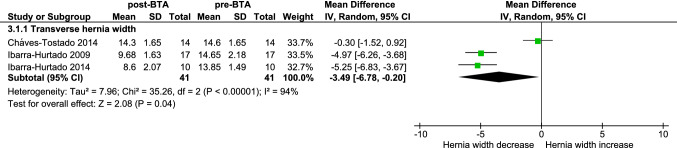
Forest plot of the difference in transverse hernia defect width in centimeters before and after BTA (without PPP). From the study by Ibarra-Hurtado et al. [7], only the patients in which CT imaging is used to measure abdominal wall defects are analyzed

Meta-analysis of three studies [7, 22, 28] that investigate the effect of BTA on transverse hernia width shows that BTA significantly decreases the hernia width by 3.5 cm (95% CI 0.2–6.8, *p* = 0.04). Study results display high heterogeneity (*I*^2^ = 94%) (Fig. 3).

### Effect of BTA + PPP on muscle- and hernia dimensions

Two studies on ventral hernia patients [31, 35] investigate the combined effect of BTA and PPP. They find a mean increase in length of the lateral abdominal wall of 3.1 cm per side for ventral hernia patients also undergoing PPP. Two studies report contradicting outcomes regarding transverse hernia width. One study [25] finds a non-significant reduction of 1.0 cm (*p* = 0.455), while another study [35] reports a significant increase in hernia width of 5.0 cm (*p* = 0.015). Two studies find a significant reduction of loss of domain after the combined treatment of BTA and PPP. Expressing loss of domain as described by Tanaka et al. [36], one study [25] reports a reduction from 29.1 to 14.1% (*p* = 0.001), and another study [24] a reduction from 28.1 to 22.8% (*p* < 0.01).

### Surgical data and clinical outcome

Surgical data and clinical outcomes of the 19 studies that investigate BTA in ventral hernia patients are presented in Table 3. Described surgical techniques are open repair [7, 15, 17, 22, 23, 25, 26, 28–31, 35], laparoscopic or laparoscopic-assisted repair [16, 20, 21], and totally extracorporeal minimally invasive repair [27]. Thirteen of nineteen studies [15–17, 19–21, 23, 25, 27–31] describe the use of mesh in all repairs, whereas in three studies [18, 22, 35], meshes are used in selected patients only. Sixteen studies describe performed CST. In these studies, 48% (IQR 25.3–69.5) of patients underwent a CST (specific types and percentages are presented in Table 3).

**Table 3:** Surgical data and clinical outcomes

References	Type repair	Component separation technique	Mesh repair	Fascial closure	Follow-up (months)	Recurrence (%)
Studies investigating BTA specifically for ventral incisional hernias						
Blaha [19]	Open hernia repair with bilateral retrorectus release	TAR 36.8%^a^ TAR 40.9%^b^	100%	100%	n.r.	n.r.
Bueno-Lledó [25]	Open hernia repair	ACS 57% TAR 32%	100%	97%	34.5	8%
Bueno-Lledó [15]	Open hernia repair	0%^a^ ACS 100%^b^	100%^a^ 100%^b^	100%^a^ 95%^b^	19.6^a,b^	0%^a^ 5%^b^
Catalan-Garza [26]	Open hernia repair	ACS 75%	n.r.	78%	24	11%
Chan [27]	Totally extracorporeal minimally invasive	n.r.	100%	100%	18.3	0%
Cháves-Tostado [28]	Open hernia repair	Unspecified 21%	100%	78%	16	0%
Deerenberg [18]	n.r.	ACS 35%, TAR 24%, combination 3%^a^ ACS 25%, TAR 15%, combination 6%^b^	97%^a^ 96%^b^	92%^a^ 81%^b^	14^a^ 29^b^	9%^a^ 12%^b^
Elstner [20]	Laparoscopic or laparoscopic-assisted	Limited endoscopic central ext. oblique release 19%	100%	100%	19	0%
Elstner [16]	Laparoscopic or laparoscopic-assisted	n.r.	100%	100%	24	0%
Farooque [21]	Laparoscopic or laparoscopic-assisted	n.r.	100%	n.r.	n.r.	n.r.
Hernández López [29]	Open hernia repair	Unspecified 25%	100%	100%	12	n.r.
Ibarra-Hurtado [7]	Open hernia repair	Unspecified 50%	n.r.	100%	9.1	0%
Ibarra-Hurtado [22]	Open hernia repair	53%^c^	24%	100%	49	0%
Kohler [33]	Open hernia repair	Endoscopic ACS 15%, TAR 24%	n.r.	n.r.	12	6%
Nielsen [30]	Open hernia repair	Endoscopic ACS 14%, ACS 11%, TAR16%	100%	100%	1	n.r.
Palmisano [23]	Open hernia repair	0%	100%	100%	n.r.	2.6%
Tashkandi [35]	Open hernia repair	Unspecified 62%	54%	100%	n.r.	n.r.
Yurtkap [31]	Open hernia repair	ACS 70%, TAR 9%, ACS or TAR 13%	100%	82%	19.5	14%
Zendejas [17]	Open hernia repair (45%) laparoscopic repair (55%)	Unspecified 18%^a^ Unspecified 8%^b^	100%^a^ 100%^b^	41%^a^ 36%^b^	15.6^a^ 18.4^b^	9%^a^ 9%^b^

*n.r.* not reported, *ACS* anterior component separation, *TAR* transversus abdominis release

^a^Represents the group of patients with BTA

^b^Represents the group of patients without BTA

^c^Only the vertical incision of the external oblique fascia lateral to the linea semilunaris and dissection of the plane between the external oblique muscle and internal oblique muscle were needed, with no further incision being required

**Fig. 4:**
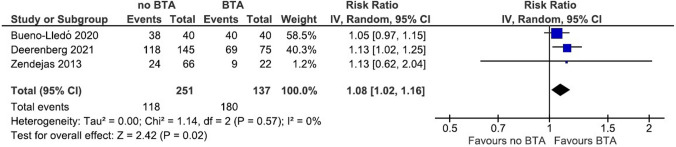
Forest plot of studies that directly compare fascial closure rate between patients with and without BTA pretreatment. Studies that combine the use of BTA and PPP are not included

Meta-analysis of three studies [15, 17, 18], which directly compare ventral hernia patients with and without BTA pretreatment, shows that BTA (without PPP) significantly increases fascial closure rate [RR 1.08 (95% CI 1.02–1.16, *I*^2^ = 0%, *p* = 0.02)] (Fig. 4).

In 14 studies [7, 16–18, 20–23, 25–30], which describe 420 BTA pretreated ventral hernia patients (without PPP), the median preoperative defect width is 13.9 cm (IQR 11.4–14.6), and fascial closure is achieved in 100% (IQR 88.5–100). During a median follow-up of 19.0 months (IQR 11.6–24.0), their median hernia recurrence rate is 0% (IQR 0–9).

## Comparative studies

### Ventral hernia

In a prospective study, Bueno-Lledo et al. [15] include 80 patients with a large midline incisional hernia (11–17 cm). Forty patients were pretreated with BTA followed by retrorectus repair, without CST, 4 weeks later. The other 40 patients did not receive BTA and underwent modified anterior CST followed by onlay mesh repair. Baseline demographics do not differ between both groups. Fascial closure is achieved in all 40 patients treated with BTA and in 38 patients of the control group (*p* = 0.372). The only significantly different outcome between both groups is the number of patients with wound dehiscence requiring reoperation, being lower in the BTA group (0 vs 4, *p* = 0.031).

In a propensity-score matched study, Deerenberg et al. [18] retrospectively investigate the fascial closure rate in patients with large ventral hernia with (*n* = 75) and without (*n* = 145) BTA. Patients from both groups were matched according to BMI, hernia width, and loss of domain. Fascial closure is achieved significantly more often in patients treated with BTA (92% vs 81%, *p* = 0.036).

In the retrospective case-controlled study by Zendejas et al. [17], 22 patients with ventral incisional hernia that had received preoperative BTA are matched with 66 controls, based on age, sex, BMI, history of hernia recurrence, and type of repair (open vs. laparoscopic). Patients treated with BTA required significantly less opioid analgesia on postoperative day 2 and 5, and reported significantly less pain on day 2 and 4. No differences in postoperative wound complications and hernia recurrence are observed.

Blaha et al. [19] specifically investigate the analgesic effect of BTA in patients undergoing open ventral hernia repair. Retrospectively, 19 patients in which BTA is injected bilateral into the oblique- and rectus muscles under direct visualization during surgery are compared to 22 controls. No significant difference is observed in postoperative pain scores nor use of morphine equivalent up to 5 days.

### Open abdomen

The double-blinded RCT by Zielinski et al. [14] describes 46 patients with an open abdomen following damage-control laparotomy (DCL). On average 1.8 days after DCL, the lateral abdominal wall muscles are injected with either BTA or normal saline. No differences in cumulative 10-day fascial closure rate nor length of hospital stay were observed. Also, throughout the first 5 days following DCL, there was no difference in morphine equivalent use.

## Discussion

In this systematic review and meta-analysis, summary evidence of 23 studies comprising 995 patients is presented. Generally, either 500 units of Dysport^®^ or 200–300 units of Botox^®^ is injected at 3–5 locations bilaterally in all three muscles of the lateral abdominal wall, about 4 weeks prior to surgery. Although technical aspects differ substantially among studies, it is fair to say that BTA pretreatment seems safe, based on the fact that not a single major complication has been reported. Meta-analyses indicate that BTA pretreatment of ventral hernia patients provides a significant elongation of the lateral abdominal wall of 3.2 cm on each side, resulting in 6.3 cm total elongation. A heterogeneous decrease in transverse hernia width was found (*I*^2^ = 94%). Furthermore, meta-analysis shows that BTA pretreatment in ventral hernia patients significantly increases the chance to achieve fascial closure.

In 2020, Deerenberg et al. published a comprehensive clinical perspective paper using data of ten studies (*n* = 398 patients) [8]. Just like previous reviews, they advocate the use of BTA prior to ventral hernia as a safe and easy to perform promising technique, but underline the fact that currently no consensus exists on how to select patients. Also in 2020, Wegdam et al. published a systematic review of 14 studies that describes the use of BTA in patients with a ventral hernia [37]. Their primary outcome is abdominal wall compliance, defined as elongation of the lateral abdominal wall. From data of four studies, they report a median elongation of the lateral abdominal muscles of 4.0 cm. As medians may overestimate the true effect, we performed a random- effects meta-analysis of the same four studies and observed a pooled overall effect of 3.2 cm elongation on each side. Distinctive from previously published studies is that the present systematic review includes all studies that describe the injection of BTA into the lateral abdominal muscles, regardless of the study population or concomitant use of PPP. Doing so, we provide a more comprehensive and unique overview pointing out similarities and important differences in the technical aspects and treatment regimens that are currently being used.

First of all, only seven studies state a specific indication to select patients for BTA treatment. This can be explained by the fact that this new application of BTA has been introduced just recently, yet makes comparison between studies difficult and underscores the need for standardization. In addition, which outcome measure best reflects the additional value of BTA is not well established. Intuitively, a decrease in transverse hernia width and increase in length of the lateral abdominal wall may feel as a positive result. The only study investigating the effect of BTA on muscle thickness, however, measured in a horizontal plane rather than perpendicular to the muscle fibers, and thus does not reflect the effect of muscle relaxation accurately. How changes in muscle dimensions truly affect surgical- and postoperative outcomes is unknown.

Another important finding of the present study is the high (100%) median fascial closure rate in patients with a ventral hernia. We should acknowledge that the majority of included studies provide poor quality of evidence and that fascial closure depends on numerous different factors, such as muscle and tissue quality, hernia size, loss of domain, and surgical technique. With this in mind, two studies are particularly interesting as they investigate fascial closure rates in ventral hernia patients, with and without BTA pretreatment, while reporting preoperative hernia width and surgical technique. Bueno-Lledo et al. [15] describe 80 patients with large ventral hernia (12–18 cm). Forty patients were pretreated with BTA and underwent open repair about 4 weeks later. The other 40 did not receive BTA and underwent open CST. Noticeable is that in all 40 BTA pretreated patients, primary closure is achieved without requiring CST. Deerenberg et al. [18] have investigated fascial closure rate in a patient cohort with large ventral hernia, treated with (*n* = 75) and without (*n* = 145) BTA. Both groups are matched according to BMI and defect size (mean hernia width 14.1 cm). They have found that fascial closure is achieved significantly more in the BTA group compared to the non-BTA group (92% vs 81%, *p* = 0.036). Because the BTA group also underwent surgical CST significantly more often (61% vs 47%, *p* = 0.042), it remains uncertain to what degree the higher fascial closure rate is explained by the use of BTA. The potential benefits of BTA were underlined by our pooled analysis of three studies comparing patients with and without BTA pretreatment, showing a significant increase in fascial closure rate (RR 1.08, 95% CI 1.02–1.16). As further prospective studies investigating the need for surgical CST and fascial closure rate in comparable patient groups with and without BTA treatment are currently lacking, the true effect of BTA on these outcomes requires further investigation.

The results of this systematic review point out several other items that warrant further research. First, comparative studies investigating the ideal type of BTA and its dosage have not been performed. Preclinical data suggest a conversion factor of 1:3 for Botox^®^ to Dysport^®^. The efficacy of both is comparable; however, the commonly used dose of Dysport^®^—perceived as optimal—comes at a slightly lower price than the commonly used dose of Botox^®^ [38]. With the currently available data, it is near impossible to assess an optimal dosage. The only study investigating a lower dosage of Botox^®^ (100 units instead of 200–300) reports on 14 patients with a mean hernia defect of 14.6 cm. Fascial closure was achieved in 78%, which could be interpreted as a suboptimal effect resulting from a lower dosage of BTA.

Second, whether or not all three lateral muscles should be injection remains questionable. The only study investigating the selective injection of two muscles compared to the standard injection of three muscles found no difference in hernia dimensions on CT. Fascial closure rates were 100% in both groups, but without mentioning the need for or type of surgical CST. Not injecting the transversus abdominis muscle can thus be considered, as the transversus abdominis muscle has an important role in truncal stability. Moreover, if a transversus abdominis release (TAR)—transecting the transversus abdominis muscle—is planned anyway, preoperative injection of that muscle can be omitted.

Third, the effect of different time intervals between injection and surgical repair on the degree of muscle paralysis and therewith abdominal wall pliability is not investigated yet. Based on the mechanism of action, BTA treatment approximately 4 weeks prior to surgery is generally accepted.

Finally, three studies hypothesize that BTA pretreatment may have an analgesic effect in the postoperative period, and have investigated the effect of BTA on postoperative pain levels and use of pain medication. In a randomized trial, in which patients with an open abdomen were injected with BTA or a placebo, no difference in use of postoperative analgesia on day 10 was observed [14]. Two other studies compare ventral hernia patients after BTA to a control group, but both report that BTA is injected at the same day as surgery in most patients. Because the paralyzing effect starts around 2 days after injection with a maximum effect 2–4 weeks later, the design of all three studies is inappropriate to determine the true analgesic (and muscle relaxation) effect of BTA.

The main strength of present study is that all studies investigating the use of BTA in abdominal wall reconstruction are included, regardless of clinical scenario. Because we analyzed studies that describe the use of additional PPP separately, the clinical scenario will reflect daily clinical practice for many centers. The results from comparing data of largely poor-quality observational studies should be interpreted in the light of potential selection- and publication bias. It is recommended against creating a funnel plot for visual inspection of asymmetry when less than ten studies are included for a specific outcome, and thus, we were unable to detect or discard publication bias.

In patients with very large ventral hernias, the need for surgical CST may not always be avoided by BTA. However, fascial closure does become more likely. In 16 studies describing patients with large and complex ventral hernias, the median fascial closure rate is 100%, and pooled analysis of comparative studies indicates that BTA improves fascial closure. Furthermore, a reduced sheer stress on the abdominal wall repair for some months may result in better healing and thereby fewer recurrences. The currently described BTA treatment regimens differ substantially and require standardization for the comparison of outcomes in future research. Randomized trials (such as: NCT04419844) and prospective studies are therefore needed to determine which type of BTA, dosage, number of injections, injected muscle, and timing with respect to the surgical repair are favorable.

## Conclusion

The injection of botulinum toxin A in the lateral abdominal wall muscles is safe without major procedural complications. The technical aspects, treatment regimens, as well as patient selection for botulinum toxin A differ substantially and require standardization. Bilateral pretreatment in hernia patients significantly elongates the lateral abdominal wall muscles, making fascial closure over mesh during surgical hernia repair more likely.

## Supplementary Information

Below is the link to the electronic supplementary material.

Supplementary file1 (DOCX 13 KB)

Supplementary file2 (DOCX 45 KB)

## Data Availability

Not applicable.
