# Supplementary material for: A systematic review and meta-analysis of technical aspects and clinical outcomes of botulinum toxin prior to abdominal wall reconstruction
Source: Hernia. 2021 Sep 21;25(6):1413–25. doi: 10.1007/s10029-021-02499-1 (PMC8613151; doi:10.1007/s10029-021-02499-1)
Supplement: Supplementary file 1 — Supplementary file1 (DOCX 13 KB) [file 10029_2021_2499_MOESM1_ESM.docx]

**Supplementary Information 2**

("Botulinum Toxins"[Mesh] OR "Clostridium botulinum"[Mesh] OR "onabotulinum toxin A" [Supplementary Concept] OR "abobotulinumtoxinA" [Supplementary Concept] OR "incobotulinumtoxinA" [Supplementary Concept] OR (("Neurotoxins"[Mesh] OR "Neurotoxins" [Pharmacological Action] OR neurotox*[tiab]) AND ("Clostridium"[Mesh] OR clostrid*[tiab])) OR botulinum*[tiab] OR (botuli*[tiab] AND toxin*[tiab]) OR botox*[tiab] OR onabotulin*[tiab] OR abobotulinumtoxin*[tiab] OR onaBoNT-A[tiab] OR incobotulin* [tiab] OR BTA[tiab] OR BTX[tiab] OR BTXA[tiab] OR BoNT A[tiab] OR BoNT serotype A[tiab] OR BoNT B[tiab] OR BoNTF[tiab] OR BoNT F[tiab] OR BoNT serotype F[tiab] OR evabotulinum*[tiab] OR disport[tiab] OR azzalure[tiab] OR meditoxin[tiab] OR neuronox[tiab] OR oculinum[tiab] OR vistabel[tiab] OR neurotoxin*[tiab] OR bocouture[tiab] OR nt201[tiab] OR oculinum[tiab] OR prosigne[tiab] OR reloxin[tiab] OR xeomin[tiab] OR myobloc[tiab] OR myoblock[tiab] OR neurobloc[tiab] OR rimabotulin*[tiab] OR exoenzyme C3[tiab] OR bacterial toxin C3[tiab] OR onaclostox[tiab] OR rimabotulinum[tiab] OR chemical component seperation*[tiab] OR chemical component relaxation*[tiab]) AND ("Hernia, Abdominal"[Mesh] OR "Incisional Hernia"[Mesh] OR "Herniorrhaphy"[Mesh] OR hernia*[tiab] OR abdominal wall[tiab] OR abdominal hernia*[tiab] OR ventral hernia*[tiab] OR incisional hernia*[tiab] OR herniorrhaph*[tiab] OR hernioplast*[tiab])
