# Supplementary material for: A systematic review and meta-analysis of technical aspects and clinical outcomes of botulinum toxin prior to abdominal wall reconstruction
Source: Hernia. 2021 Sep 21;25(6):1413–25. doi: 10.1007/s10029-021-02499-1 (PMC8613151; doi:10.1007/s10029-021-02499-1)
Supplement: Supplementary file 2 — Supplementary file2 (DOCX 45 KB) [file 10029_2021_2499_MOESM2_ESM.docx]

**Supplementary Information 3**

| **Quality assessment, following the Newcastle-Ottowa scale(13)** | | | | | | | | | | |
| --- | --- | --- | --- | --- | --- | --- | --- | --- | --- | --- |
|  |  |  |  |  |  |  |  |  |  |  |
| Study (year) | Representa-tiveness of the exposed cohort | Selection of the non-exposed cohort | Ascertainment of exposure | Demonstration that outcome of interest was not present at start of study | Comparability of cohorts on the basis of the design or analysis controlled for confounders | Assessment of outcome | Follow-up long enough for outcomes to occur | Adequacy of follow-up of cohorts | Stars | Quality  assessment |
| Blaha  2020(19) | B* | A* | A* | n.a. | B* | B* | B | D | 5 | Poor |
| Bueno-Lledó  2020(25) | A* | n.a. | A* | n.a. | n.a. | B* | A* | A* | 5 | Poor |
| Bueno-Lledó  2020(15) | B* | A* | A* | n.a. | B* | B* | A* | B* | 7 | Good |
| Catalan-Garza  2020(26) | B* | n.a. | A* | n.a. | n.a. | B* | A* | C | 4 | Poor |
| Chan  2019(27) | C | n.a. | A* | n.a. | n.a. | B* | A* | A* | 4 | Poor |
| Cháves-Tostado  2014(28) | B* | n.a. | A* | n.a. | n.a. | B* | A* | A* | 5 | Poor |
| Deerenberg  2021(18) | A* | A* | A* | n.a. | B* | B* | A* | B* | 7 | Good |
| Elstner  2017(20) | C | n.a. | A* | n.a. | n.a. | B* | A* | A* | 4 | Poor |
| Elstner  2020(16) | C | n.a. | A* | n.a. | B* | B* | A* | A* | 5 | Poor |
| Farooque  2016(21) | C | n.a. | A* | n.a. | n.a. | B* | A* | A* | 4 | Poor |
| Hernandez López  2016(29) | B* | n.a. | A* | n.a. | n.a. | B* | A* | A* | 5 | Poor |
| Hipolito Canario  2020(34) | C | n.a. | A* | n.a. | n.a. | B* | B | D | 2 | Poor |
| Ibarra-Hurtado  2009(7) | B* | n.a. | A* | n.a. | n.a. | B* | B | A* | 4 | Poor |
| Ibarra-Hurtado  2014(22) | B* | n.a. | A* | n.a. | n.a. | B* | A* | A* | 5 | Poor |
| Kohler  2020 (33) | C | n.a. | A* | n.a. | n.a. | B* | A* | C | 3 | Poor |
| Nielsen  2020(30) | A* | n.a. | A* | n.a. | n.a. | B* | B | A* | 4 | Poor |
| Palmisano  2019(23) | A* | n.a. | A* | n.a. | n.a. | B* | B | D | 3 | Poor |
| Tang  2020(24) | C | n.a. | A* | n.a. | n.a. | B* | A* | B* | 4 | Poor |
| Tashkandi  2021(35) | B* | n.a. | A* | n.a. | n.a. | B* | B | D | 3 | Poor |
| Yurtkap  2020(31) | A* | n.a. | A* | n.a. | C | B* | A* | A* | 5 | Poor |
| Zendejas  2013(17) | B* | A* | A* | n.a. | A* | B* | A* | A* | 7 | Good |
| Zielinski  2013(32) | C | n.a. | A* | n.a. | n.a. | B* | A* | D | 3 | Poor |
| Zielinski  2016(14) | C | A* | A* | n.a. | A* | B* | A* | A* | 6 | Fair |
|  |  |  |  |  |  |  |  |  |  |  |
| Good quality: 3 or 4 stars in selection domain AND 1 or 2 stars in comparability domain AND 2 or 3 stars in outcome/exposure domain.  Fair quality: 2 stars in selection domain AND 1 or 2 stars in comparability domain AND 2 or 3 stars in outcome/exposure domain.  Poor quality: 0 or 1 star in selection domain OR 0 stars in comparability domain OR 0 or 1 stars in outcome/exposure domain.  *n.a.* not applicable | | | | | | | | | | |

| **Quality assessment, following the revised Cochrane risk of bias tool(12)** | | | | | | |
| --- | --- | --- | --- | --- | --- | --- |
| Study (year) | Randomization process | Deviations from intended intervention | Missing data | Outcome measurement | Selection of reported results | Overall risk of Bias assessment |
| Zielinski  2016(14) | Low RoB | Low RoB | Low RoB | Low RoB | Some concerns regarding RoB | Some concerns |
| *RoB* Risk of Bias | | | | | | |
